# Ciliopathies in Complex Congenital Heart Disease: Molecular Genetics, Embryologic Mechanisms and Clinical Implications

**DOI:** 10.3390/genes17050584

**Published:** 2026-05-19

**Authors:** Maria Felicia Gagliardi, Emanuele Micaglio, Angelo Micheletti, Sara Benedetti, Andrea Giordano, Baldassare Maria Alonzo, Giulia Guglielmi, Diana Gabriela Negura, Alessandro Giamberti, Massimo Chessa

**Affiliations:** 1 Faculty of Medicine and Surgery, Milano-Bicocca University, 20126 Milan, Italy; 2 Pediatric and Adult Congenital Heart Centre, IRCCS Policlinico San Donato, San Donato Milanese, 20097 Milan, Italy; 3 Arrhythmia and Electrophysiology Department, IRCCS Policlinico San Donato, San Donato Milanese, 20097 Milan, Italy; 4 Congenital Cardiac Surgery Unit, IRCCS Policlinico San Donato, San Donato Milanese, 20097 Milan, Italy; 5 Faculty of Medicine and Surgery, Vita-Salute San Raffaele University, 20132 Milan, Italy

**Keywords:** congenital heart disease, ciliopathies, primary cilia, cardiac morphogenesis, embryogenesis, developmental signaling pathways

## Abstract

**Background/Objectives:** Congenital heart malformations (CHDs) are not rare diseases, and, in many cases, their pathogenic mechanisms are well established. Several conotruncal defects are associated with genetic syndromes such as DiGeorge syndrome and RASopathies, reflecting shared developmental pathways affecting cardiac outflow tract formation. However, even common CHDs may occur within complex syndromic contexts, making early diagnosis essential for optimal management. This review aims to provide a unifying framework linking ciliary dysfunction to CHD phenotypes. **Methods:** We performed an integrative narrative review of genetic, experimental, and developmental studies focusing on the role of primary and motile cilia in cardiac morphogenesis. Particular attention was given to signaling pathways regulated by cilia and their contribution to disease phenotypes. **Results:** Emerging evidence indicates that primary and motile cilia act as central regulators of cardiac development, integrating morphogen gradients and mechanical cues into transcriptional programs. Dysfunctions in ciliary structure or signaling are increasingly recognized as important contributors to selected complex CHD phenotypes, particularly in syndromic forms and laterality-associated defects. This cilia-centered model may help explain part of the phenotypic heterogeneity observed in CHD and highlights shared mechanisms across distinct clinical entities. **Conclusions:** Understanding cilia-dependent mechanisms provides a unifying conceptual framework linking genetic defects to disrupted morphogenesis. This perspective may refine disease interpretation and support future development of precision diagnostics and pathway-informed therapeutic strategies in CHD.

## 1. Introduction

### Ciliopathies: Extracardiac Manifestations and Cardiac Involvement

Congenital heart disease (CHD) represents one of the most frequent developmental anomalies associated with genetic and syndromic conditions, often characterized by marked phenotypic heterogeneity [1,2,3,4]. In this context, ciliopathies have emerged as a relevant group of inherited disorders caused by structural or functional defects of primary and motile cilia, which play essential roles in embryonic development and tissue homeostasis [5,6,7,8,9]. Evidence from murine mutagenesis and large-scale association studies has demonstrated a pivotal role for cilia in congenital heart disease (CHD), with many implicated genes related to ciliary structure, signaling pathways, and vesicular trafficking [5,6,7,8,9,10,11,12]. Notably, several of these genes are involved in left–right axis patterning, suggesting that defects in cardiac laterality contribute significantly to CHD pathogenesis [11,13,14]. Consistently, patients with CHD show a high prevalence of ciliary dysfunction and de novo variants in cilia-related pathways, supporting the fact that selected CHD phenotypes may be interpreted within a ciliopathy-related framework [1,2,3,5,6,10,15].

**Figure 1:**
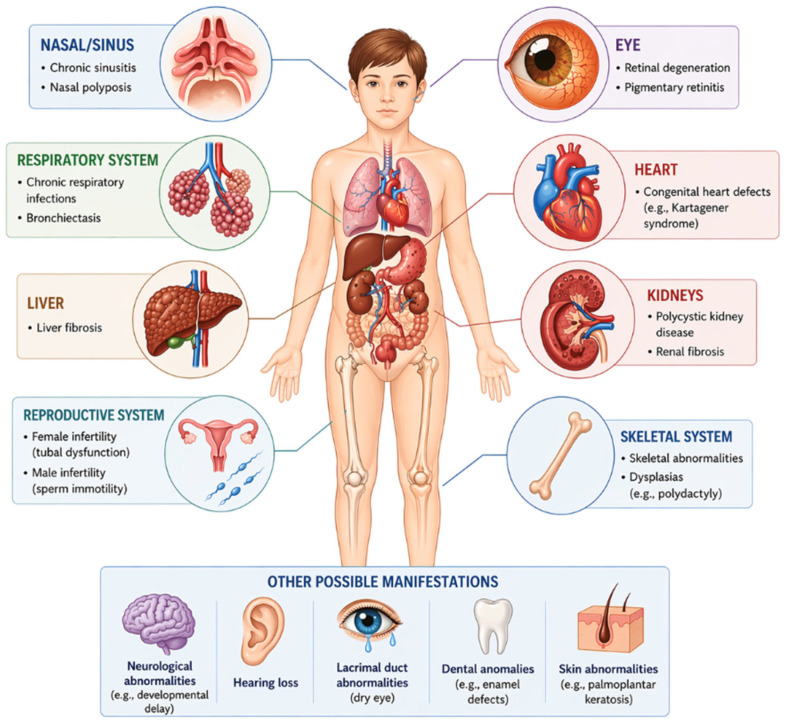
Extracardiac manifestations of Ciliopathies.

Due to the ubiquitous distribution of cilia, ciliopathies present with a broad spectrum of extracardiac manifestations affecting multiple organ systems (Figure 1) [8,15]. Common features include renal disease (e.g., polycystic kidney disease, nephronophthisis), retinal dystrophy, and progressive vision loss, particularly in Bardet–Biedl and Joubert syndromes [16]. Neurological abnormalities such as cerebellar malformations and neurodevelopmental delay are frequent, along with hepatic fibrosis, skeletal anomalies, polydactyly, and endocrine dysfunction [7,8,9,15]. Disorders of motile cilia, such as primary ciliary dyskinesia, are characterized by impaired mucociliary clearance, chronic respiratory disease, and recurrent infections; Kartagener syndrome combines these features with situs inversus due to defective left–right patterning [5,6].

Cardiac involvement in ciliopathies is variable but particularly relevant in conditions affecting left–right patterning and developmental signaling pathways [5,6,7,8,15]. In addition to laterality defects, structural heart anomalies are observed in syndromes such as Bardet–Biedl, Joubert, and Meckel–Gruber, where disruption of cilia-dependent pathways (e.g., Hedgehog, Wnt/PCP, Notch) impairs cardiac morphogenesis, including septation and outflow tract development [5,6,7,8,9,11]. The coexistence of cardiac and extracardiac features highlights the role of cilia as integrators of developmental signaling networks and supports the classification of ciliopathies as systemic disorders requiring multidisciplinary management [6,7,15,17].

To contextualize these defects, an overview of normal cardiovascular development is essential. The mammalian heart is a four-chambered organ that supports separate pulmonary and systemic circulations, with functional asymmetry dependent on correct left–right axis establishment during embryogenesis [13,14,18]. Proper cardiac morphogenesis includes chamber formation (LA, RA, LV, RV), septation, outflow tract division into the aorta and pulmonary artery, and valve development to ensure unidirectional blood flow [18]. Disruptions in these processes, as seen in CHD, lead to severe hemodynamic consequences and high neonatal morbidity and mortality without intervention [1,2,3]. Understanding the genetic and developmental basis of CHD is therefore critical for improving diagnosis and therapeutic strategies [1,2,3,12,19].

## 2. Experimental Evidence Supporting Ciliary Function in Cardiac Development

Evidence supporting the role of cilia in congenital heart disease is strongest for laterality defects, heterotaxy, and selected complex CHD phenotypes and derives from complementary experimental approaches, including in vivo animal models, in vitro and ex vivo systems, and human genetic studies. In vivo studies, particularly forward genetic screens and targeted mouse mutants, have demonstrated a significant enrichment of cilia-related genes among the causes of congenital heart disease. These models show that disruption of ciliary structure or signaling leads to cardiac looping defects, conotruncal malformations, septation defects, and heterotaxy, closely recapitulating human CHD phenotypes [11,13,14]. Complementary in vitro and ex vivo studies have provided mechanistic insights into these defects, demonstrating that primary cilia function as signaling platforms regulating Hedgehog, Wnt/planar cell polarity (PCP), and Notch pathways, as well as mechanosensory signaling required for Nodal activation [5,6,7,9,12,19]. These findings highlight how defective receptor trafficking, impaired signal transduction, or altered cytoskeletal organization directly affect cardiomyocyte polarity, proliferation, and differentiation. Finally, human genetic studies have identified rare inherited and de novo variants in cilia-related genes in patients with complex CHD, particularly in syndromic conditions such as ciliopathies and heterotaxy. Together, these data support a causal link between ciliary dysfunction and several cardiac malformations, while the strength of evidence varies across specific CHD subtypes [1,2,3,6,10].

## 3. Structure and Function of Primary Cilia

Primary cilia are slender, microtubule-based organelles protruding from the apical surface of most vertebrate cells, including cardiomyocytes and endocardial cells. They function as cellular “antennae,” detecting extracellular chemical and mechanical signals and translating them into intracellular responses that guide cell behavior during heart development [5,6,7,8,9].

**Figure 2:**
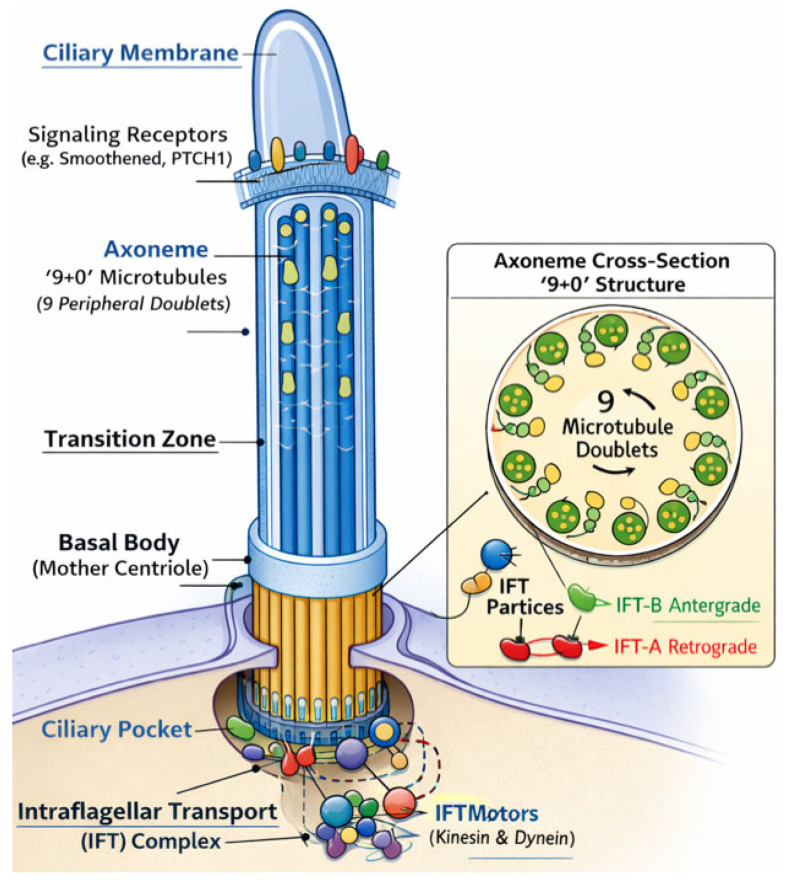
Structure of primary cilia.

Each cilium is composed of a basal body, which anchors the cilium to the cell and organizes microtubules; an axoneme, typically arranged in a 9 + 0 microtubule pattern; and a specialized ciliary membrane enriched in receptors and signaling proteins (Figure 2) [7,8,9]. Because the cilium is apically positioned and protrudes into the extracellular space, it is ideally suited to sample morphogen gradients and mechanical forces, providing spatial information that individual cells can translate into transcriptional programs [7,8,9].

**Figure 3:**
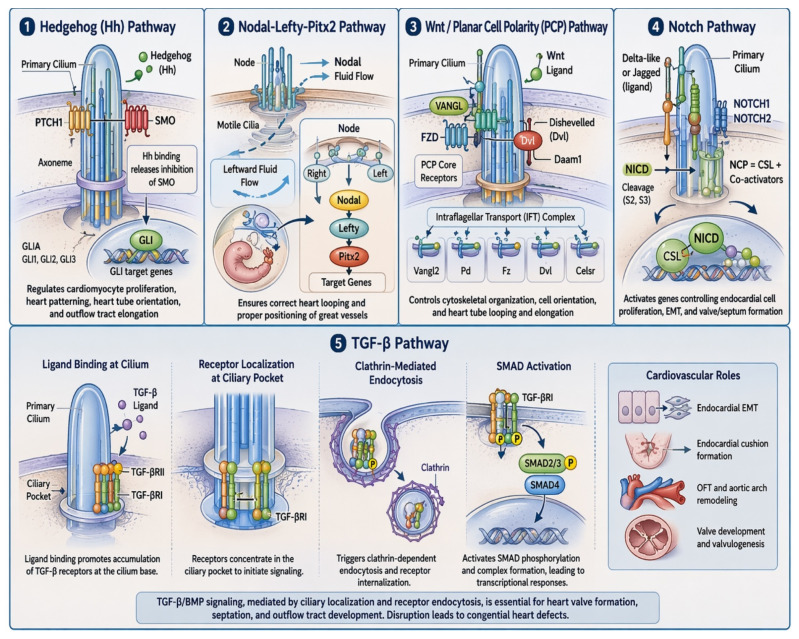
Major primary cilia–dependent signaling pathways regulating cardiac development. In order, the Hedgehog (Hh), Nodal–Lefty–Pitx2, Wnt/Planar Cell Polarity (PCP), Notch and TGF beta pathways.

Primary cilia act as signaling hubs controlling key developmental pathways (Figure 3):

1. **Hedgehog (Hh) Pathway:** Cilia detect Hedgehog proteins via the PTCH1 receptor. When Hedgehog binds, it activates SMO, which triggers GLI transcription factors in the nucleus. This pathway regulates cardiomyocyte proliferation, patterning of heart chambers, proper orientation of the heart tube and outflow tract elongation [5,6,7].
2. **Nodal-Lefty-Pitx2 Pathway:** Motile cilia in the embryonic node create a directional fluid flow that determines the left-right axis of the body. This flow induces the expression of Nodal on the left side, which activates Lefty and Pitx2 transcription factors. This pathway ensures that the heart loops correctly and that the great vessels (aorta and pulmonary artery) are positioned appropriately [13,14,15].
3. **Wnt/Planar Cell Polarity (PCP) Pathway:** Primary cilia orchestrate Wnt/PCP signaling by localizing core PCP receptors, such as Frizzled (FZD) and Van Gogh-like (VANGL), on the ciliary membrane. When Wnt ligands bind these receptors, they trigger downstream effectors (Disheveled, Daam1) that organize the cytoskeleton within the cell. This controls the directional orientation of cardiomyocytes, guiding how cells elongate, align, and intercalate. Coordinated cell orientation drives elongation and rightward looping of the heart tube, aligning the ventricles and outflow tracts. If cilia are defective or PCP signaling is impaired, cells fail to polarize correctly [6,7].
4. **Notch Pathway:** Cilia act as scaffolds to concentrate Notch receptors (NOTCH1/2) and their ligands (Delta/Jagged) at the apical surface of endocardial cells. The Notch intracellular domain (NICD) is cleaved and translocates to the nucleus. In the nucleus, NICD binds to the DNA-binding transcription factor CSL (CBF1/RBPJ) and recruits co-activators to form the Notch transcriptional complex (NCP). This complex activates gene expression programs that control endocardial cell proliferation, differentiation, and epithelial-to-mesenchymal transition (EMT). Proper signaling ensures formation and remodeling of endocardial cushions, guiding atrial and ventricular septation and valve development [5,6,7].
5. **TGF-β Pathway**: Recent studies have demonstrated a critical role for primary cilia in facilitating TGF-β signaling. Ligand binding promotes the accumulation of TGF-β receptors at the base of the cilium, within a specialized region known as the ciliary pocket [20]. This receptor localization triggers clathrin-mediated endocytosis, culminating in downstream activation of SMAD phosphorylation [20]. The essential involvement of TGF-β/BMP signaling in congenital heart disease (CHD) has been well characterized through both in vitro and in vivo studies in chick and mouse embryos, as well as analyses of knockout mouse models [21,22,23]. These investigations reveal that TGF-β/BMP signaling orchestrates multiple aspects of cardiovascular morphogenesis, including regulation of endocardial epithelial-to-mesenchymal transition (EMT) and endocardial cushion formation [24,25]. For example, early growth of endocardial cushions, essential for the establishment of functional cardiac valves, depends on BMP signaling in cardiac neural crest cells via BMPRIA receptors [26]. The role of Tgfb2 in outflow tract (OFT) and aortic arch remodeling is evidenced by the perinatal lethality of Tgfb2 knockout mice, which exhibit double outlet right ventricle and interrupted aortic arch [27]. Disruption of TGF-β/BMP signaling likely contributes significantly to valvular defects observed in mouse models with impaired clathrin-mediated endocytosis and receptor recycling, including mutations in Ap2b1, Dnm2, Ap1b1, Snx17, LRP1, and LRP2. These mutants display OFT malalignment and endocardial cushion anomalies, phenotypes comparable to those observed in models with defective TGF-β/BMP signaling [21,22,23]. Similarly, defects in ciliary structure within the endocardial cushions may perturb cilia-mediated TGF-β/BMP signal transduction required for normal valvulogenesis. For instance, mutations in Cc2d2a, a component of the ciliary transition zone, result in selective cilia loss in the atrioventricular (AV) cushions, while the outflow cushions remain unaffected. Correspondingly, these mutants exhibit AV valve malformations, with preservation of normal outflow valve development.

Together, these pathways allow cilia to translate external signals into coordinated cell behaviors that shape the heart. Importantly, these signaling cascades do not act in isolation but interact through shared ciliary mechanisms, including receptor trafficking, compartmentalized signal transduction, cytoskeletal organization, and mechanosensory inputs. Disruption of these pathways may contribute to recognizable patterns of complex congenital heart disease, although the precise hierarchy and context-dependent integration of these signals remain incompletely defined [5,6,7,15,16].

## 4. Detailed Ciliary Signaling During Cardiac Embryogenesis

**Figure 4:**
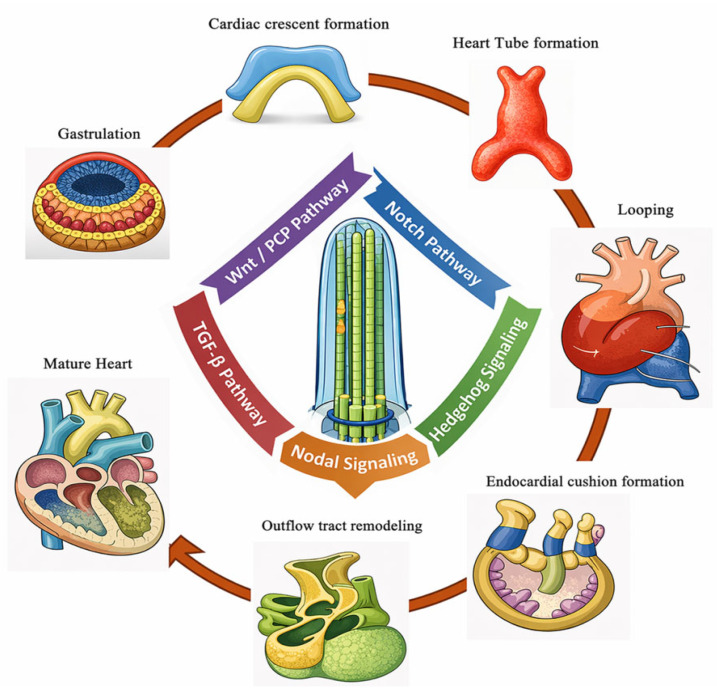
Ciliary Signaling During Cardiac Embryogenesis.

Cilia operate at distinct stages and locations during heart development (Figure 4), and each stage contributes uniquely to the formation of the heart [12,28]:

**Gastrulation (E6.5–E7.5 in mice; week 3 in humans):** Primary cilia appear on mesodermal progenitor cells in the anterior lateral plate mesoderm. Here, cilia sense gradients of Hedgehog and Wnt ligands, directing cardiomyocyte specification, mesoderm patterning, and establishing the early left-right axis. Dysfunction at this stage can mispattern cardiac progenitors, leading to downstream anomalies [5,6,7,18].

**Cardiac crescent formation (E7.5–E8.0 in mice; week 3–4 in humans):** The cardiac crescent forms from bilateral heart fields. Primary cilia on cardiomyocytes and endocardial cells transduce Hedgehog and Notch signals, coordinating proliferation, differentiation, and alignment of heart field cells [5,6,7,18].

**Linear heart tube formation and looping (E8.0–E9.0 in mice; week 4 in humans):** Motile cilia in the embryonic node generate leftward fluid flow, triggering Nodal-Lefty-Pitx2 signaling that establishes proper left-right asymmetry [13,14,15,16]. Simultaneously, primary cilia on the forming heart tube sense Hedgehog and Wnt/PCP signals to regulate directional looping, myocardial proliferation, and chamber alignment [5,6,7].

**Endocardial cushion formation and septation (E9.5–E12.5 in mice; week 5–8 in humans):** Primary cilia concentrate on endocardial cushion cells, where they integrate Hedgehog, Wnt/PCP, and Notch signals. This guides atrial and ventricular septation, valvulogenesis, and alignment of the outflow tract [5,6,7,20,21,22,23,24,25].

**Outflow tract remodeling and great vessel formation (E10.5–E14.5 in mice; week 6–10 in humans):** Cilia on endothelial and migrating neural crest cells orchestrate rotation and septation of the aorticopulmonary septum, ensuring proper formation of the aorta and pulmonary artery [5,6,7,26,27].

By mapping ciliary function to each embryonic stage, it becomes clear that cilia are not static but relocate and adapt their signaling roles according to the morphogenetic needs of the developing heart. This demonstrates that cilia act as spatiotemporal regulators of morphogenesis rather than static organelles [6,7,8].

The unexpected discovery that a significant proportion of genes identified in congenital heart disease (CHD) screens are associated with cilia or cilia-related processes was particularly striking, given that these studies were entirely phenotype-driven. This finding highlights the central role of cilia biology in regulating cardiovascular development and underscores its contribution to CHD pathogenesis [5,6,7,8,11].

Primary cilia in the developing heart were first described by electron microscopy in embryos of multiple species, including chicken, rabbit, mouse, and lizard [29]. These early observations revealed that cilia were present exclusively in non-mitotic cardiomyocytes or myoblasts, whereas in the mature heart, cilia were primarily detected in fibroblasts [29].

Subsequent studies in mouse embryos demonstrated that primary cilia are widely distributed throughout the early heart tube at embryonic day (E) 9.5 [30]. As development progresses to E12.5, cilia persist in specific cardiac regions, including the atria and trabeculated myocardium, indicating a sustained role during chamber maturation [30,31]. In addition, cilia are present in the atrial endocardial layer and are particularly enriched in the endocardial cushion mesenchyme and epicardium, suggesting a role in valve formation and myocardial–epicardial interactions [9,30,31].

A growing body of evidence demonstrates that cilia functions as signaling hubs that coordinate multiple pathways essential for cardiac morphogenesis [5,6,7,9]. Among these, Hedgehog (Shh), TGF-β/BMP, and Wnt signaling pathways are especially prominent regulators of cardiovascular development and are strongly implicated in the etiology of CHD [5,6,7,20,21,22,23].

Consistent with this, large-scale mouse genetic screens for CHD have identified several genes linked to Hedgehog signaling, including Sufu, Fuz, Tbc1d32, and Kif7, further supporting the importance of cilia-mediated signaling in heart development [11].

## 5. Genetic Basis of Ciliary Dysfunction

### 5.1. Core Genetic Regulators of Ciliary Signaling and Trafficking

When discussing the genetic basis of ciliary dysfunction in CHD, it is important to distinguish between genes with well-established causal roles in syndromic ciliopathies and genes whose contribution to isolated or complex CHD is supported mainly by experimental models or associative evidence. This distinction is particularly relevant because evidence levels differ substantially between human cohorts and animal studies.

At the molecular level, the integrity of cilia-dependent signaling pathways relies on a network of genes that regulate ciliogenesis, ciliary architecture, and protein trafficking within the cilium. Disruption of these genes compromises the ability of primary cilia to coordinate key developmental pathways—including Hedgehog, Wnt/planar cell polarity (PCP), Notch, Nodal and TGF beta signaling—thereby perturbing the tightly controlled processes of outflow tract septation, rotation, and alignment described above [5,6,7,13,14].

The ***BBS1–BBS12*** genes, mutated in Bardet–Biedl syndrome, encode components of the BBSome, a multiprotein complex essential for trafficking proteins to and from the ciliary membrane. These genes have well-established causal roles in human ciliopathies characterized by multisystem involvement, including renal and retinal disease [15,16]. Cardiac defects have been reported in these patients, although they are variable and not consistently present. The BBSome regulates the localization of key signaling receptors, including Smoothened (SMO) and Patched1 (PTCH1) in the Hedgehog pathway, as well as Frizzled (FZD) and Van Gogh-like (VANGL) receptors involved in Wnt/PCP signaling [6,16]. Although VANGL proteins are critical for both cardiac and neural tube development, variants in VANGL genes have not been consistently identified in patients with isolated congenital heart defects. In addition, BBSome dysfunction may indirectly affect Notch signaling by altering receptor organization at the ciliary membrane. Mutations in BBS genes impair receptor trafficking and cell polarity in established ciliopathy models, and have been associated with defects in outflow tract alignment and conotruncal septation [6,11,16].

***CEP290*** encodes a transition zone protein that functions as part of the ciliary gate, regulating selective protein entry and exit. This gene is a well-recognized cause of human ciliopathies, particularly those involving retinal degeneration and renal disease [15]. While its role in ciliary signaling is well established, its specific contribution to CHD phenotypes is less consistently defined and is supported largely by experimental and functional studies [6,7,12]. Disruption of CEP290 primarily affects Hedgehog signaling by impairing GLI transcription factor activation and downstream transcriptional responses [6,7,12].

***OFD1*** encodes a protein localized at the basal body and centrioles that is required for ciliogenesis. Mutations in OFD1 cause syndromic ciliopathies in humans, including oral-facial-digital syndrome, and provide strong evidence for its role in ciliary function [5,6,7]. However, its association with CHD is more variable and often occurs within broader syndromic contexts. OFD1 contributes to the regulation of Hedgehog signaling, with secondary effects on additional cilia-dependent pathways such as Wnt and Notch [5,6,7].

***MKS1*** and ***TMEM67*** (Meckelin) encode transition zone proteins that contribute to the structural organization of the ciliary gate and regulate the molecular composition of the cilium. These genes are well-established causes of severe ciliopathies, such as Meckel–Gruber syndrome, and play a central role in early embryonic patterning [15]. Their involvement in Nodal and Hedgehog signaling is critical for establishing left–right asymmetry, and defects in these genes are strongly associated with laterality abnormalities in both human disease and animal models [13,14,15,16].

***PKD1*** and ***PKD2*** encode polycystin-1 and polycystin-2, which form a mechanosensitive calcium channel complex localized in motile cilia of the embryonic node. These proteins detect the leftward fluid flow generated by nodal cilia and initiate intracellular calcium signaling required for activation of the Nodal pathway. Mutations in *PKD1* or *PKD2* impair flow sensing, disrupt calcium signaling, and result in defective left–right axis specification [13,14,15].

Importantly, these genes do not belong to single signaling pathways but instead regulate fundamental aspects of ciliary structure, trafficking, and mechanosensation. Through these functions, they simultaneously modulate multiple signaling cascades—including Hedgehog, Wnt/PCP, Notch, Nodal and TGF beta pathways—highlighting the role of primary cilia as integrative signaling hubs during cardiac development [4,5,6,7,9]. This multifunctional role explains why mutations in ciliary genes often lead to overlapping yet phenotypically heterogeneous cardiac defects, depending on the specific pathway and developmental stage affected.

### 5.2. Microtubule-Based Intraflagellar Transport

**Figure 5:**
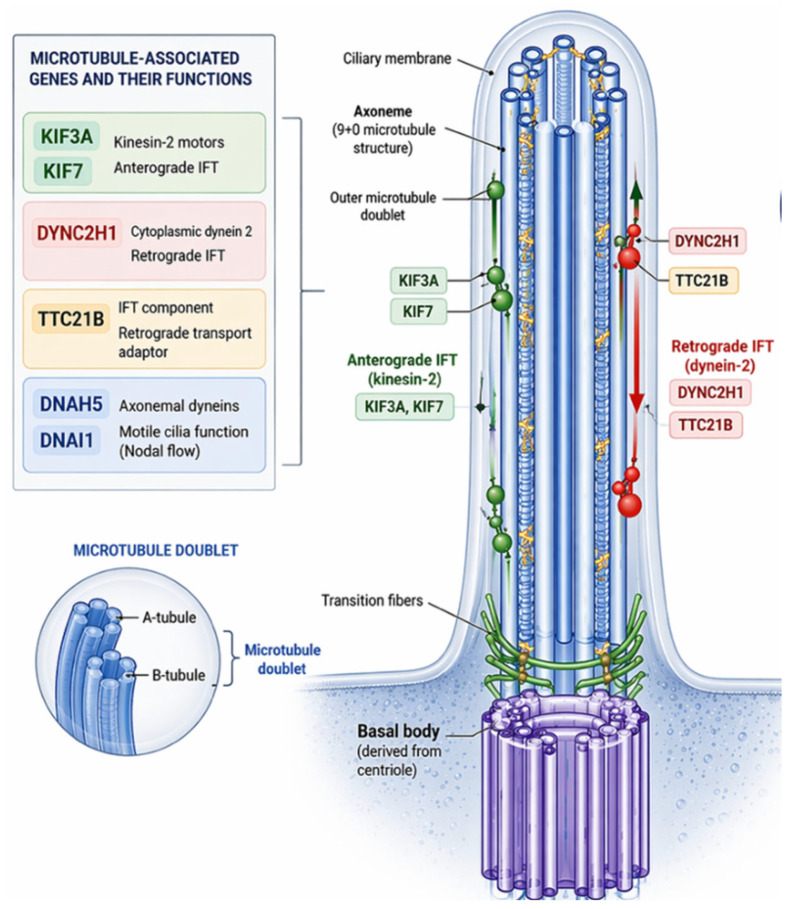
Microtubule associated genes.

In addition to genes regulating ciliary trafficking and signaling, the integrity of the microtubule cytoskeleton represents a fundamental determinant of ciliary function. The ciliary axoneme is composed of highly organized microtubule doublets that provide both structural support and a platform for intraflagellar transport (IFT), a bidirectional trafficking system essential for the proper localization of signaling receptors and downstream effectors [5,6,7,8,9,32]. Key components of this system include kinesin motor proteins encoded by *KIF3A* and *KIF7* genes, which mediate anterograde transport, and dynein motors such as *DYNC2H1*, which regulate retrograde trafficking along axonemal microtubules [32,33] (Figure 5). These genes, particularly *KIF3A* and *KIF7*, have been extensively characterized in large-scale mutagenesis screens and experimental models, providing mechanistic insights into ciliary function, although their direct contribution to human CHD remains less well established [7,11]. Mutations affecting these microtubule-associated proteins disrupt ciliary assembly and signaling competence, leading to defective coordination of Hedgehog, Wnt/planar cell polarity (PCP), and Nodal pathways.

Additional genes involved in microtubule-dependent transport, such as *TTC21B*, further highlight the importance of retrograde IFT in modulating signal transduction within the cilium. Disruption of these mechanisms impairs GLI processing in the Hedgehog pathway and alters cellular polarity cues, ultimately affecting cardiomyocyte alignment and outflow tract morphogenesis [5,6,7,32,34]. Moreover, defects in axonemal dynein genes associated with motile cilia, including *DNAH5* and *DNAI1*, compromise the generation and sensing of nodal flow, thereby interfering with calcium-dependent activation of Nodal signaling and leading to abnormalities in left–right axis specification [13,14,15,16,32].

Collectively, these observations demonstrate that microtubule-associated genes are not merely structural components but are essential regulators of ciliary signaling. Their dysfunction provides a mechanistic link between impaired intracellular transport, defective signal integration, and the development of a broad spectrum of cardiac phenotypes, ranging from isolated laterality defects to complex conotruncal malformations [5,6,7,11,15,32].

## 6. Ciliopathy-Associated Complex Congenital Heart Disease

Complex congenital heart diseases (CHDs) associated with ciliopathies may arise from perturbations in cilia-dependent signaling pathways that regulate cardiac morphogenesis, leading to distinct yet mechanistically interconnected phenotypes [5,6,7,10,11]. Notably, the genetic architecture of these conditions is often more complex than simple monogenic inheritance. In a subset of patients, CHD may result from oligogenic or digenic mechanisms, in which heterozygous variants in two different genes within the same biological pathway—such as those regulating axonemal structure or intraflagellar transport—act synergistically to produce a disease phenotype [7,10,11].

This concept is well illustrated by classical ciliopathies such as Bardet–Biedl syndrome, where mutational burden across multiple BBS genes can modulate disease expression and severity [16]. Similar mechanisms have also been proposed in other ciliopathies, including Joubert syndrome and Meckel–Gruber syndrome, both of which primarily present with multisystem involvement but may include cardiac defects in a subset of patients [6,7,15,35,36].

Although a comprehensive discussion of all ciliopathies is beyond the scope of this review, it is important to highlight these two syndromes due to their relevance to cardiac development. In Joubert syndrome, congenital heart defects are relatively uncommon but have been reported, including bicuspid aortic valve, atrial septal defects, and, in some cases, left ventricular outflow tract obstruction such as aortic stenosis [15,35]. In contrast, Meckel–Gruber syndrome, a severe and often lethal ciliopathy, is more frequently associated with complex cardiac malformations, including hypoplastic left heart syndrome and rare anomalies such as cor triatriatum, reflecting profound disruption of early embryonic patterning and cilia-mediated signaling [6,7,15,36].

These observations further support the notion that ciliary dysfunction affects cardiac development through shared molecular pathways, while the final phenotype depends on the combined genetic context and the specific developmental processes affected, reinforcing the concept of ciliopathies as a continuum rather than discrete clinical entities [5,6,7].

Importantly, although ciliopathies may be associated with a broad spectrum of cardiac malformations—including left-sided obstructive lesions such as hypoplastic left heart syndrome—the most consistent and mechanistically well-characterized defects involve abnormalities of outflow tract development and left–right patterning. Different congenital heart defects therefore reflect disruption of distinct cilia-dependent developmental processes, including septation, alignment, rotation, and laterality. The following section focuses on selected representative phenotypes that exemplify these mechanisms.

**Tetralogy of Fallot (TOF)** is one of the prototypical conotruncal defects that has been associated, in experimental and genetic studies, with perturbations in cilia-dependent signaling pathways. It is characterized by four cardinal features: malalignment of the outflow septum, pulmonary stenosis, overriding aorta, and right ventricular hypertrophy. Developmentally, TOF results from abnormal anterior deviation and asymmetric growth of the conotruncal septum, processes tightly regulated by Hedgehog and Wnt/planar cell polarity (PCP) signaling. Disruption of Hedgehog signaling impairs second heart field proliferation and outflow tract elongation, whereas defective PCP signaling alters cellular polarity and directional tissue remodeling, leading to misalignment of the ventricles and great vessels. In addition, impaired cilia-dependent Notch signaling compromises epithelial-to-mesenchymal transition (EMT) and endocardial cushion formation, further contributing to septal and valvular defects [5,6,7,21,22,23]. At the molecular level, these alterations are frequently associated with mutations in genes such as *BBS1–BBS12*, *CEP290*, and *OFD1*, which affect ciliary structure, receptor trafficking, and signal transduction, particularly within Hedgehog and PCP pathways [6,11,12].

**Transposition of the great arteries (TGA)** is defined by ventriculo-arterial discordance, in which the aorta arises from the right ventricle and the pulmonary artery from the left ventricle, resulting in parallel rather than crossed circulations. Developmentally, this defect arises from failure of normal spiralization of the aorticopulmonary septum, primarily due to defects in left–right patterning. Impaired motile cilia function at the embryonic node disrupts the generation and sensing of directional fluid flow, leading to defective activation of the Nodal–Lefty–Pitx2 signaling cascade [13,14,15,16]. Concurrently, altered Hedgehog signaling may impair proper outflow tract rotation, further contributing to vascular misalignment. At the molecular level, these abnormalities are associated with mutations in genes such as *MKS1*, *TMEM67*, and *PKD1/2*, which regulate transition zone integrity and cilia-mediated mechanosensory calcium signaling required for Nodal pathway activation [14,15,16].

**Double outlet right ventricle (DORV)** is defined by the origin of both great arteries predominantly from the right ventricle, resulting in impaired ventriculo-arterial alignment. Developmentally, DORV arises from defective coordination of outflow tract alignment and septation, processes regulated by Hedgehog, Wnt/PCP, and Notch signaling. At the molecular level, these alterations are associated with mutations in genes such as *BBS1–BBS12*, *CEP290*, *OFD1*, and *MKS1/TMEM67*, which disrupt ciliary trafficking, structural integrity, and transition zone function [6,11,12].

Importantly, these phenotypes reflect distinct developmental failures: TOF primarily arises from conotruncal septation defects, TGA from impaired outflow tract rotation, and DORV from defective alignment between ventricles and great arteries.

**Heterotaxy syndromes** are characterized by abnormal left–right organ arrangement and are strongly associated with defects in motile cilia function at the embryonic node. These conditions arise from impaired generation or sensing of leftward fluid flow, processes essential for activation of the Nodal signaling pathway. Disruption of cilia-driven flow leads to defective calcium signaling and failure of left–right axis specification, resulting in a broad spectrum of cardiac anomalies, including atrial isomerism, abnormal venous return, and complex outflow tract defects [10,13,14,15,16]. At the molecular level, these abnormalities are linked to mutations in genes such as *PKD1*, *PKD2*, *MKS1*, *TMEM67*, *BBS1–BBS12*, *CEP290*, and *OFD1*, which collectively regulate ciliary structure, mechanosensation, and signaling during early embryogenesis [6,11,15,16].

These observations support the concept that ciliary dysfunction does not act through isolated pathways but rather through the coordinated disruption of interconnected signaling networks governing cardiac morphogenesis, ultimately resulting in a spectrum of complex and interrelated cardiac phenotypes [5,6,7,9]. The formation and alignment of the outflow tract depend on coordinated interactions among neural crest cells, second heart field progenitors, and cilia-mediated signaling pathways [5,6,7,18]. In this context, Hedgehog signaling promotes second heart field proliferation and outflow tract elongation, Wnt/PCP signaling governs cellular polarity and directional tissue remodeling, Notch signaling regulates endocardial cushion formation and septation, and Nodal signaling establishes left–right asymmetry [5,6,7,15,16,21,22,23]. Together, these pathways orchestrate key morphogenetic processes, including conotruncal ridge formation, spiralization of the aorticopulmonary septum, wedging of the outflow tract, and closure of the interventricular septum [18,21,22,23].

Disruption of these mechanisms leads to distinct developmental outcomes: defective conotruncal ridge formation results in persistent truncus arteriosus; asymmetric ridge development leads to anterior septal deviation and Tetralogy of Fallot; failure of septal spiralization results in transposition of the great arteries; and impaired ventriculo-arterial alignment gives rise to double outlet right ventricle [5,6,7,18].

## 7. Clinical Implications of Ciliary Dysfunction in Complex CHD

An important concept emerging from recent studies is that genes involved in ciliary structure and function can be associated with both isolated congenital heart defects (CHDs) and syndromic forms with extracardiac manifestations, highlighting the pleiotropic nature of cilia-related genes and their role as central regulators of multiple developmental pathways [5,6,7,10,15]. This paradigm suggest that, in selected cases, the traditional dichotomy between syndromic and non-syndromic CHD and instead supports the existence of a phenotypic continuum driven by the extent and context of ciliary dysfunction. Components of cilia-dependent signaling networks—including pathways interacting with Notch, Hedgehog, and Wnt/planar cell polarity (PCP) signaling—contribute to a wide spectrum of phenotypes ranging from isolated cardiac malformations to complex multisystem disorders [5,6,7,21,22,23].

Notably, this concept is not restricted to classical ciliopathy genes. While genes such as *BBS1–BBS12*, *CEP290*, *OFD1*, *MKS1*, and *TMEM67* are well-established causes of syndromic ciliopathies (e.g., Bardet–Biedl and Meckel syndromes), they have also been implicated in non-syndromic CHD, reinforcing the idea of shared molecular mechanisms underlying diverse clinical presentations [6,7,11,15]. In parallel, genes involved in major developmental pathways—such as *NOTCH1* and its ligand *JAG1*, as well as cytoskeletal regulators like *FLNA*—can give rise to both isolated cardiac defects (e.g., bicuspid aortic valve or left-sided obstructive lesions) and syndromic conditions with broader phenotypic involvement, supporting the concept of variable expressivity within cilia-regulated signaling networks [5,6,7,21,22,23].

Importantly, additional cilia-related genes involved in left–right patterning and mechanosensory signaling—such as *PKD1*, *PKD2*, and transition zone components including *MKS1* and *TMEM67*—further exemplify this continuum, as they may manifest either as isolated laterality defects or as part of complex heterotaxy syndromes with multisystem involvement [13,14,15,16].

Within this spectrum, laterality defects may present in a highly subtle and clinically underrecognized form.

Dextrocardia may represent the sole clinical manifestation of an underlying ciliary dysfunction, occurring in the absence of conotruncal defects or other structural cardiac anomalies. This phenotype likely reflects a selective perturbation of left–right axis specification without significant impairment of subsequent cardiac morphogenesis [6,7,13,14,15,16].

From a clinical perspective, this observation has critical implications. The identification of dextrocardia—even when isolated—should prompt consideration of an underlying ciliopathy. Consequently, genetic evaluation should be considered in patients presenting with dextrocardia, particularly when additional extracardiac features, family history, or laterality anomalies are present [1,2,3,6,7,10,15].

It is also noted that clinical pictures of children affected by dextrocardia might worsen over time requiring a specific clinical follow-up not rarely with the contribution of different specialists.

More broadly, the recognition of ciliary dysfunction as a contributing mechanism in selected forms of complex CHD has important implications for diagnosis, risk stratification, and therapeutic development [1,6,7]. To translate these concepts into clinical practice, Table 1 summarizes the main clinical clues suggestive of ciliary dysfunction in patients with CHD and their diagnostic and management implications.

As highlighted in Table 1, the recognition of specific clinical patterns suggestive of ciliary dysfunction may inform diagnostic strategies.

From a diagnostic standpoint, the identification of mutations in cilia-related genes—including *BBS1–BBS12*, *CEP290*, *OFD1*, *MKS1*, *TMEM67*, *PKD1*, and *PKD2*—supports the potential integration of targeted ciliary gene panels and whole-exome or whole-genome sequencing into the evaluation of patients with conotruncal defects, heterotaxy, or syndromic CHD [1,2,3,6,7,10,15]. This is particularly relevant in individuals with extracardiac features suggestive of ciliopathies, where a unifying genetic diagnosis can directly inform clinical management.

From a developmental and prognostic perspective, understanding which signaling pathways are disrupted provides insight into phenotypic variability and disease severity [6,7]. For instance, defects primarily affecting Nodal signaling and left–right axis specification are more commonly associated with heterotaxy and complex cardiac malformations involving abnormal venous return, whereas disruption of Hedgehog and Wnt/PCP signaling is more frequently linked to conotruncal defects such as tetralogy of Fallot and double outlet right ventricle [5,6,7,13,14,15,16]. This pathway-based framework may contribute to improving genotype–phenotype correlations and, in the future, help refine risk prediction models in CHD [1,2,3,6,7].

Furthermore, the role of cilia as integrative signaling hubs highlights potential avenues for future targeted therapeutic strategies, although these approaches remain largely experimental [6,7,9]. Although current CHD management remains predominantly surgical, emerging experimental approaches suggest that modulation of key developmental pathways—such as Hedgehog or Notch—may influence cardiac progenitor behavior, tissue remodeling, and repair [5,6,7,21,22,23]. Advances in stem cell-derived systems and cardiac organoids further provide platforms to model patient-specific ciliary defects and explore personalized therapeutic strategies in preclinical settings [12,13,14,15,16,17].

Finally, the recognition of ciliary dysfunction as a shared mechanism across multiple CHD phenotypes underscores the importance of multidisciplinary clinical evaluation, integrating cardiology, medical genetics, nephrology, and developmental biology [1,6,7,15]. Early identification of ciliopathy-associated CHD—including subtle presentations such as isolated dextrocardia—may enable proactive surveillance for extracardiac manifestations and improve long-term outcomes through coordinated care. Collectively, these insights position ciliary biology as a promising framework for future precision medicine approaches in congenital heart disease, with implications extending from prenatal diagnosis to lifelong clinical management [1,2,3,6,7,10,15].

## 8. Future Directions and Open Questions

Despite significant advances, several key questions remain regarding the role of cilia in cardiac development and congenital heart disease. First, the precise mechanisms by which different ciliary sub compartments (basal body, transition zone, and axoneme) selectively regulate distinct signaling pathways remain incompletely understood [6,7,8,9]. Second, it is still unclear how disruptions in ciliary signaling result in highly variable phenotypes, even among patients carrying mutations in the same gene, suggesting the contribution of genetic modifiers and environmental influences [3,6,7,10].

Another major challenge is to define how ciliary signaling is dynamically regulated across different stages of cardiac development and within distinct cardiac cell populations, including cardiomyocytes, endocardial cells, and neural crest cells [5,6,7,18]. While animal models have provided fundamental mechanistic insights, translating these findings to human disease remains complex, underscoring the need for advanced human-specific systems such as induced pluripotent stem cells and cardiac organoids [12,17,19].

Finally, the therapeutic potential of targeting cilia-dependent signaling pathways remains largely unexplored. A deeper understanding of pathway-specific contributions to disease phenotypes may enable the development of precision medicine strategies aimed at modulating key signaling nodes in a context-dependent manner [6,7,9]. Integration of multi-omics approaches with patient-specific organoid models will be essential to dissect pathway-specific effects and to support the development of targeted therapeutic interventions [12,17,19].

## 9. Conclusions

Ciliary biology has emerged as a fundamental determinant of cardiac development, providing a unifying framework that links genetic defects to disrupted morphogenetic processes in complex congenital heart disease [1,5,6,7]. By integrating mechanical cues and morphogen signaling, primary and motile cilia coordinate key events including left–right axis specification, cardiac looping, and outflow tract alignment. Disruption of these processes does not result in isolated anomalies but rather in a spectrum of interconnected cardiac phenotypes, often associated with multisystem involvement characteristic of ciliopathies [5,6,7,10]. Advances in genetic and experimental studies have significantly expanded our understanding of the molecular basis of cilia-related cardiac defects, highlighting the contribution of genes involved in ciliogenesis, protein trafficking, and mechanosensation [6,7]. These insights have important clinical implications and may support the integration of genomic approaches into diagnostic workflows; further studies are needed to improve genotype–phenotype correlations in patients with complex CHD [1,2,18]. Despite these advances, important challenges remain, including the need to better define the spatiotemporal regulation of ciliary signaling during cardiac development and to understand the mechanisms underlying phenotypic variability. Future research integrating human-specific models, such as induced pluripotent stem cells and organoid systems, with advanced genomic technologies will be essential to translate mechanistic insights into clinical applications [12,19]. Ultimately, a deeper understanding of cilia-dependent developmental processes will not only refine our interpretation of congenital heart disease but also pave the way for future pathway-informed therapeutic strategies and precision medicine approaches in cardiovascular care [6,7].

## Figures and Tables

**Table 1:** Clinical implications of ciliary dysfunction in congenital heart disease (CHD).

CLINICAL CLUE IN CHD PATIENT	INTERNATION	RECOMMENDED EVALUATION
**HETEROTAXY, SITUS INVERSUS, SITUS AMBIGUUS, OR DEXTROCARDIA**	Strongly suggests abnormal left–right patterning and possible motile cilia dysfunction	Perform detailed cardiac and extracardiac anatomical assessment; consider genetic testing for cilia-related genes
**CONOTRUNCAL DEFECTS, ESPECIALLY TOF, DORV, TGA, OR COMPLEX OUTFLOW TRACT MALFORMATIONS**	May reflect disruption of cilia-dependent developmental pathways involved in outflow tract alignment and septation	Consider syndromic evaluation, especially if extracardiac signs are present
**CHD ASSOCIATED WITH RENAL CYSTS, NEPHRONOPHTHISIS, OR UNEXPLAINED RENAL DYSFUNCTION**	Renal involvement is one of the most common extracardiac manifestations of ciliopathies	Add renal ultrasound and nephrology follow-up
**CHD WITH RETINAL DYSTROPHY, VISUAL IMPAIRMENT, OR ABNORMAL EYE FINDINGS**	Suggests syndromic ciliopathies such as Bardet–Biedl or Joubert syndrome	Request ophthalmologic evaluation and targeted genetic work-up
**CHD WITH CHRONIC RESPIRATORY DISEASE, RECURRENT INFECTIONS, SINUSITIS, OR BRONCHIECTASIS**	May indicate primary ciliary dyskinesia, especially when associated with laterality defects	Refer for pulmonology evaluation and diagnostic testing for motile cilia dysfunction
**CHD WITH POLYDACTYLY, SKELETAL ANOMALIES, DEVELOPMENTAL DELAY, CEREBELLAR MALFORMATIONS, OR HEPATIC FIBROSIS**	Indicates possible multisystem ciliopathy rather than isolated CHD	Activate multidisciplinary assessment including genetics, neurology, hepatology, and orthopedics as appropriate
**APPARENTLY ISOLATED DEXTROCARDIA**	May be the only visible sign of underlying ciliary dysfunction	Consider clinical context; genetic counseling and longitudinal surveillance may be appropriate when additional risk factors or extracardiac features are present
**FAMILY HISTORY OF CHD, SITUS ANOMALIES, RENAL DISEASE, RETINAL DISEASE, OR RECURRENT RESPIRATORY DISEASE**	Supports inherited or oligogenic ciliopathy-related mechanisms	Offer genetic counseling and consider cascade testing
**PRENATAL DETECTION OF COMPLEX CHD WITH EXTRACARDIAC ANOMALIES**	May indicate severe syndromic ciliopathy and affects prognosis and counseling	Recommend fetal echocardiography, detailed anomaly scan, genetic testing, and multidisciplinary prenatal counseling

## Data Availability

No new data were generated in this study. All information supporting the conclusions of this article is included within the article and its references.
